# Polarization-dependent strong coupling between silver nanorods and photochromic molecules

**DOI:** 10.3762/bjnano.9.247

**Published:** 2018-10-08

**Authors:** Gwénaëlle Lamri, Alessandro Veltri, Jean Aubard, Pierre-Michel Adam, Nordin Felidj, Anne-Laure Baudrion

**Affiliations:** 1 Light, nanomaterials and nanotechnologies (L2n), Institut Charles Delaunay (CNRS), Université de Technologie de Troyes (UTT), 12 rue Marie Curie, CS 42060, 10004 Troyes Cedex, France; 2 Colegio de Ciencias e Ingeniera, Universidad San Francisco de Quito, Quito, Ecuador; 3 Laboratoire Interfaces, Traitements, Organisation et Dynamique des Systèmes (ITODYS), UMR CNRS 7086, Université Paris-Diderot, Sorbonne Paris Cité, 15 rue Jean-Antoine de Baïf, 75205 Paris Cedex 13, France

**Keywords:** active plasmonics, photochromic molecules, plasmon, Rabi splitting, strong coupling

## Abstract

Active plasmonics is a key focus for the development of advanced plasmonic applications. By selectively exciting the localized surface plasmon resonance sustained by the short or the long axis of silver nanorods, we demonstrate a polarization-dependent strong coupling between the plasmonic resonance and the excited state of photochromic molecules. By varying the width and the length of the nanorods independently, a clear Rabi splitting appears in the dispersion curves of both resonators.

## Introduction

For decades, plasmonic systems have been extensively studied for their potential applications in many research fields. Due to their localized surface plasmon resonance (LSPR), metallic nanoparticles have been used to enhance the sensitivity of bio- or chemo-sensors [1], enhance and direct the light emitted by quantum dots or molecules [2–3], and to kill cancer cells [4]. This resonance is directly linked to the intrinsic properties of the metallic nanoparticles (depending on the geometry or the nature of the metal), which makes it difficult to easily control its spectral position. Many approaches have been explored to actively control these plasmonic properties without changing the topographic features of the nanoparticles themselves. Liquid crystals [5], thermosensitive polymers [6], transition metal dichalcogenides [7] and graphene [8] monolayers have been used for this purpose. The change in the refractive index or the doping ability of these materials allows the plasmonic system’s environment to be actively changed and the plasmonic properties to be controlled. Another way to control LSPR is to use photochromic molecules. These molecules can switch their conformation from a transparent state to a colored state by absorbing UV light and can return to their original state by a heating process [9] or by absorbing visible light [10]. Indeed, the photochromic transition is reversible and can undergo several cycles [11]. Current applications of these molecules are mainly found in macroscopic optical components, such as lenses [12] or sunglasses [13]. From the plasmonic perspective, the photochromic molecules can allow for the active control of the plasmonic resonance. The coupling between molecular exciton and plasmonic resonance can lead to weak [14] or strong coupling [15]. The latter is always observed when the plasmon or the molecular exciton presents a large oscillator strength and leads to the splitting of the main resonance. We previously demonstrated a reversible, strong coupling between silver cylindrical nanoparticles and photochromic molecules, both considered as largely damped oscillators [16]. It is of note that the main dipolar plasmonic resonance sustained by a cylindrical nanoparticle is not dependent on the in-plane incident polarization due to the symmetry center of the nanoparticle. However, a polarization-dependent control of the optical properties of a nanosource would be useful in nano-optics applications. As an example, Zhou et al. were able to fabricate a two-color hybrid nanosource by trapping different emitters in the close vicinity of a cylindrical nanoparticle [17]. The color emitted by this nanosource then depends on the incident in-plane polarization. In this context, a polarization-dependent external control of the plasmonic properties could be of prime interest for active plasmonic devices. In this work, we make use of the same protocol as in [16] and apply it to nanorods to demonstrate a polarization-dependent strong coupling between plasmonic resonances and the excited state of photochromic molecules, as well as the existence of a strong coupling regime when the plasmonic resonance matches the wavelength of the molecular transition. Theoretical calculations confirm our experimental findings.

## Experimental

**Figure 1:**
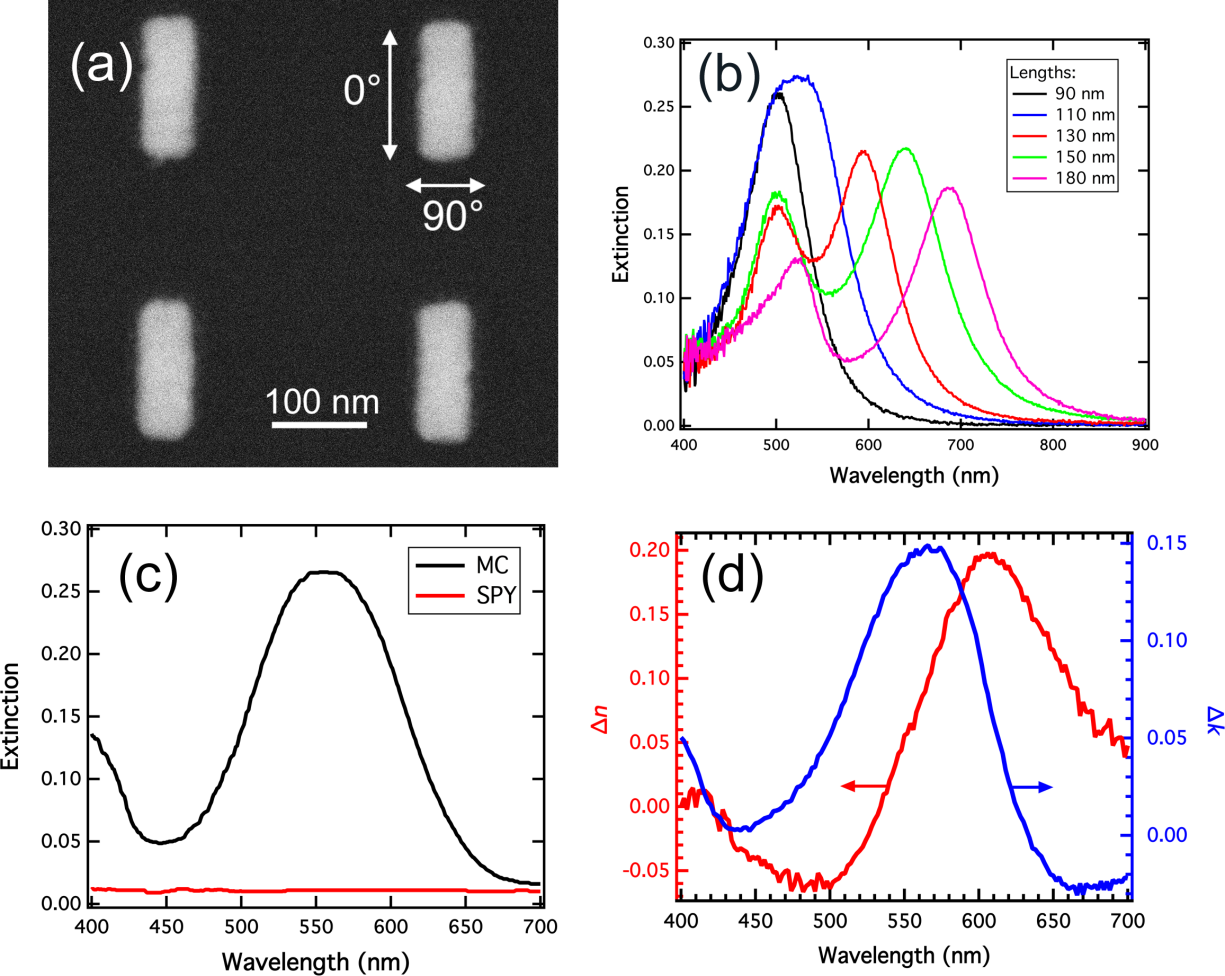
(a) SEM image of silver nanorods. The 0° and 90° polarization orientations correspond to the nanorod long and short axes, respectively. (b) Extinction spectra of silver nanorods in air. The width is 90 nm and the lengths vary from 90 to 180 nm. (c) Absorbance spectrum of a 60 nm thick active layer before (SPY) and after (MC) the photochromic transition. (d) Variations of the refractive index real and imaginary parts of the organic layer due to the photochromic transition, measured by ellipsometry.

We used standard electron-beam lithography to fabricate large arrays of silver nanorods on a glass substrate. The pitch of the arrays has been varied to keep the filling factor approximately equal to 10% and to avoid any lattice mode contribution in the optical spectra. For three different nanorod widths (70, 90 and 110 nm), the nanorod length was varied from the width value to the double width value. The height of the nanorods was fixed at 50 nm. Figure 1a shows a scanning electron microscope (SEM) image of a nanorod array recorded after the fabrication process. We used standard extinction spectroscopy to record the LSPR on each array. A halogen lamp is used to illuminate the sample from the glass side and the transmitted light is recorded through a 20× bright-field objective. The signal is then sent to a spectrometer to record extinction spectra. The Figure 1b shows typical extinction spectra recorded on five different nanorod arrays, presenting the same width (90 nm) and different lengths. In that case, the halogen lamp is not polarized and one can observe two different peaks on each spectrum, corresponding to the well-known dipolar LSPR excited on the short and the long axes of the nanorods. The photochromic molecules we used are 1′,3′-dihydro-8-methoxy-1′,3′,3′-trimethyl-6-nitrospiro[2*H*-1-benzopyran-2,2′-(2*H*)-indole] molecules (from Sigma-Aldrich). They are able to switch from their transparent spiropyran isomer (SPY) to their colored merocyanine isomer (MC) by absorbing ultraviolet light. To achieve a polymer coating, we diluted the sample in a poly(methyl methacrylate) (PMMA) solution in toluene and spin-coated them onto the sample. The phototransition is realized by illuminating the sample with a Xe lamp filtered with a 400 nm low-pass filter. The excitation lasts two minutes and the polymer film becomes violet. Indeed, the MC isomer presents an absorption maximum at 570 nm (Figure 1c). We characterized this film (without any metallic nanoparticles) by ellipsometry measurements and verified that the photochromic transition is accompanied by a high refractive index change. Figure 1d shows the variations of the refractive index real part (*n*) and imaginary part (*k*) due to the photochromic transition. One can observe that Δ*k* is maximum at 570 nm, corresponding to the absorption of the colored MC isomer. Moreover, the photochromic transition leads to a negative variation of Δ*n* for incident wavelengths below 520 nm and a positive variation for incident wavelengths above 520 nm. It is important to note that the reverse photochromic transition (from MC to SPY) can be realized either by absorbing green light (in the MC absorption band) or by heating the sample [18]. In our case, the spectroscopic measurements performed on the sample after the photochromic transition were sufficiently fast to avoid this reverse photochromic transition, which was confirmed by measuring the absorption of the MC layer before and after the measurements.

The surface plasmon resonance of the coated Ag nanorods was characterized by extinction spectroscopy before and after the photochromic transition. The incident light was polarized either along the long axis (0°) or along the short axis (90°) of the nanorods, as represented on the Figure 1a. For each polarization, all the spectra obtained on the arrays were normalized by the spectra measured nearby the nanorod arrays in order to eliminate the contribution of the molecular absorption band of the MC isomer.

## Results and Discussion

**Figure 2:**
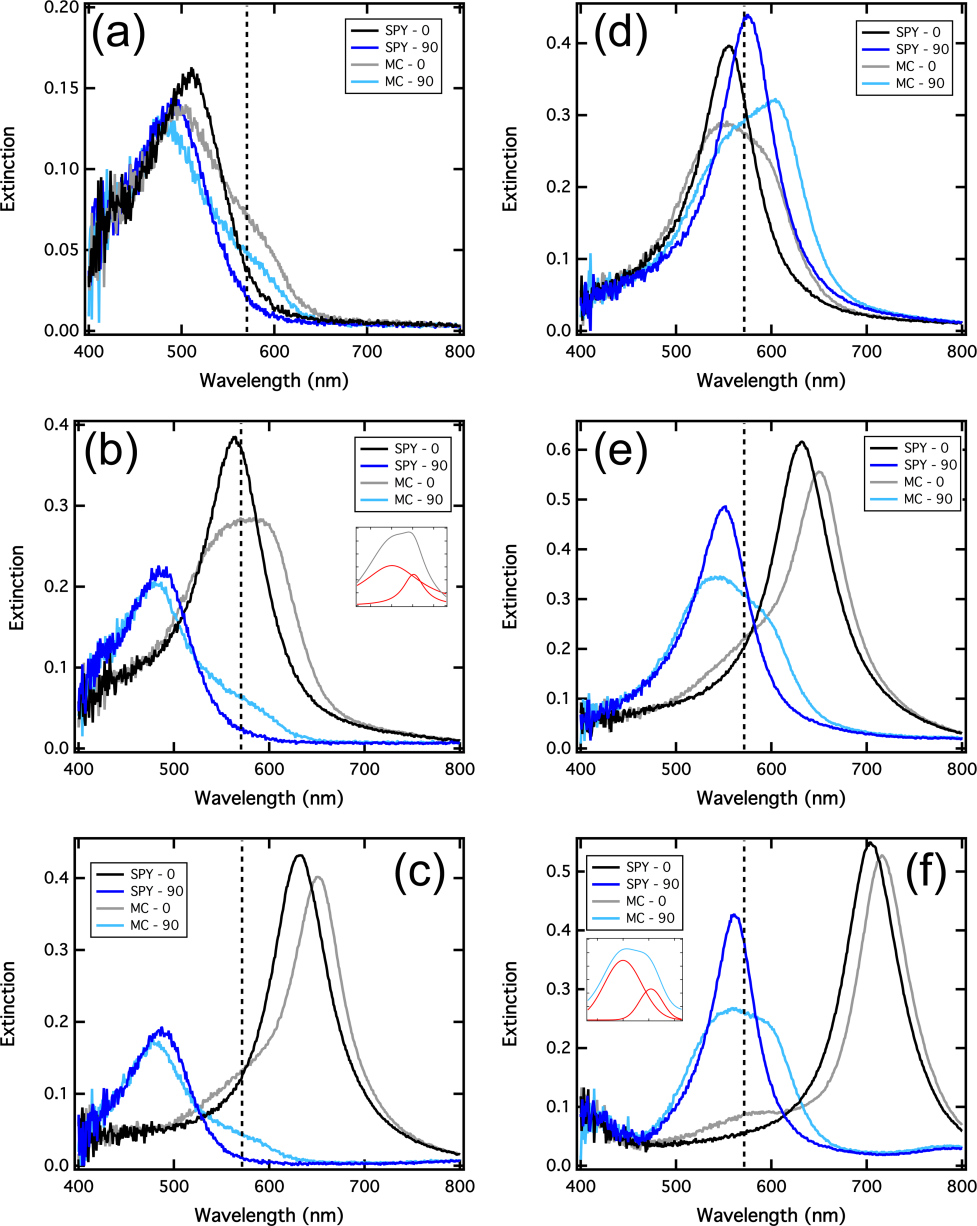
Extinction spectra of the silver nanorod arrays covered with photochromic molecules before (in SPY) and after (in MC) the photochromic transition. The 0° and 90° polarization orientations, corresponding to the excitation of the long and the short axis, are in black/grey and in dark/light blue, respectively. (a), (b) and (c) are measurements on nanorods presenting a width of 70 nm and lengths of 70, 90 and 110 nm, respectively. (d), (e) and (f) are measurements on nanorods presenting a width of 90 nm and lengths of 90, 110 and 130 nm, respectively.

Figure 2 shows the extinction spectra for nanorod widths of 70 nm (a–c) and 90 nm (d–f). In each graph, the blue curves are related to the short axis plasmonic resonances and the black curves are related to the long axis plasmonic resonances. Moreover, a vertical dashed line represents the MC absorption band at 570 nm. The influence of the photochromic transition on the spectra can be studied by comparing on one side the dark blue and the light blue curves, for the short axis resonance, and on the other side the black and the grey curves, for the long axis resonance. Firstly, one can observe in Figure 2a that before the photochromic transition (in the SPY isomer), the 70 × 70 nm nanorods display two different plasmonic resonances in the short axis (dark blue curve) and in the long axis (black curve). Indeed, the nanorod width is slightly shorter than the nanorod length and the dipolar resonance for a 0° polarization is red-shifted compared to the one for the 90° polarization. It is important to note that both resonances are located at wavelengths shorter than the MC absorption band maximum. After the photochromic transition in the MC isomer, both main resonances are blue-shifted. Moreover, a small shoulder appears near 600 nm in both the light-blue and grey curves. The 8 nm blue-shift measured for the main peak for both polarizations can be related to the negative value of the real part of the refractive index which takes place for wavelengths below 520 nm. The small shoulder comes directly from the coupling between the plasmonic resonance and the excited state of the MC molecule. In order to explore this coupling in more detail, we present the extinction spectra recorded on the nanorods in Figure 2b, presenting a width of 70 nm and a length of 90 nm. In that case, in the SPY form, the plasmonic resonance at 0° (black curve), i.e., in the nanorod long axis, coincides with the MC absorption band, whereas the plasmonic resonance at 90° (dark blue curve), i.e., in the nanorod short axis, remains as before blue-shifted compared to the MC absorption band. The photochromic transition (from the black to the grey curve) leads to a decrease of the main peak amplitude and to a clear enlargement of its spectral width. Our previous study with nanocylinders [16] allowed us to identify this behavior as a strong coupling regime, where the coincidence of the MC absorption band with the plasmonic regime leads to the formation of two distinct peaks and a so-called Rabi splitting [19]. The strong coupling regime is usually observed on high quality resonators as atoms or cavities [15]. As plasmonic resonances are low quality resonators, the strong coupling regime has been mainly studied with molecular J-aggregates, exhibiting very sharp excitonic peaks [20–21]. Even if some studies have also used rhodamine 6G and metallic nanoparticles, which are both bad resonators, they used lattice resonances and benefited from the sharp Fano-type resonance to observe the strong coupling anti-crossing behavior [22]. In our case, each resonator (the LSPR and the molecular exciton) presents a large full width at half maximum (FWHM). Indeed, the black curve of the Figure 2b gives a FWHM of 75 nm, corresponding to 300 meV. Moreover, the FWHM of the MC absorption (Figure 1c) is measured at about 130 nm, corresponding to 500 meV. The Rabi splitting, originating in the cross between the dispersion curves of the two resonant modes, is usually observable if its energy is larger than the sum of their line widths [19]. This condition implies that the Rabi oscillation period is shorter than the damping time of the plasmon and of the organic exciton. In our case, the fitted decomposition of the curves into two Lorentzian curves (inset of the Figure 2b) leads to an energy splitting of 190 meV, far lower than the 400 meV (150 + 250 meV) necessary to its observation. Although it can be attributed to a line narrowing due to the coupling [23], we think that this peak analysis is not relevant in our case as we measure the plasmonic resonance and the molecular excitons over a large number of metallic nanoparticles and molecules respectively. Indeed, the nanofabrication process does not allow us to obtain identical geometries over this large number of particles, especially for this nanorod geometry where a rounding of the corners occurs. The extinction measurements do not reflect the quality of the strong coupling between one single nanorod and the molecular exciton. Indeed, even if one single nanorod couples strongly and coherently to an ensemble of molecules, it does not mean that this coupled system can coherently couple to the neighbor coupled systems. This incoherent sum of the contributions and the averaging of the extinction signal over a large number of particles increases the resonance’s FWHM. It is worth noting that our previous results on arrays of nanocylinders allowed us to observe two distinct peaks, probably because of the geometry deviations that are minimized for nanocylinders.

Finally, Figure 2c shows the extinction spectra recorded on 70 nm wide and 110 nm long nanorods. The short axis resonance before and after the photochromic transition (blue curves) are identical to Figure 2a and 2b, but in this case, the long axis resonance in the SPY form (black curve) is located at a longer wavelength compared to the MC absorption band. The photochromic transition leads here to a red-shift of the main resonance of about 20 nm, corresponding to a positive Δ*n* of 0.16. Figure 2d–f corresponds to the extinction spectra recorded on 90 nm wide nanorods with varying lengths of 90, 110 and 130 nm, respectively. On each graph, the dark blue plasmonic resonance coincides with the MC absorption band, and the strong coupling regime can thus be observed on the transverse axis of the nanorods in the MC isomer (light blue curves). In the longitudinal plasmonic resonance in the SPY form, varying from 550 nm (Figure 2d) to 700 nm (Figure 2f), the photochromic transition leads to a strong coupling regime for the 90 nm long nanorods and to a weak coupling regime for 130 nm long nanorods.

**Figure 3:**
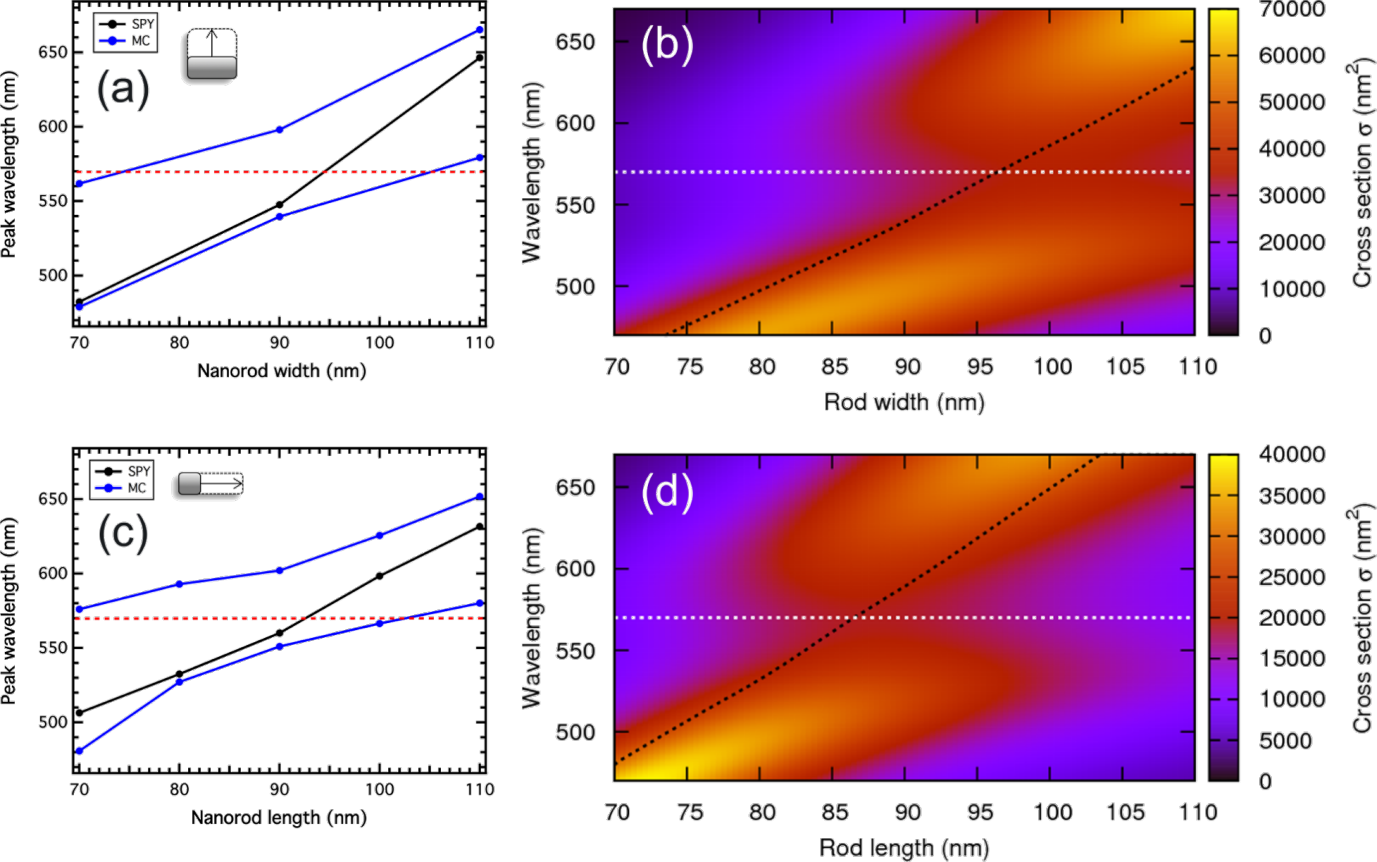
(a) Experimental spectral evolution of the transverse (90°) plasmonic peak as a function of the rod width for a fixed length of 110 nm. (b) Calculated extinction cross-section map for the corresponding ellipsoids as a function of the wavelength and the ellipsoid width. (c) Experimental spectral evolution of the longitudinal (0°) plasmonic peak as a function of the rod length for a fixed width of 70 nm. (d) Calculated extinction cross-section map for the corresponding ellipsoids as a function of the wavelength and the ellipsoid length.

This spectral analysis was performed for various nanorod geometries and we plotted the position of the plasmonic peaks as a function of the rod width and the rod length for a 110 nm fixed length and a 70 nm fixed width, respectively (Figure 3a and 3c). For each graph, the surface plasmon resonance in the SPY isomer is given by the black line whereas the blue curves show the evolution of the peaks after UV irradiation. The dashed horizontal red lines represent the position of the MC absorption band. Either for the transverse, or for the longitudinal plasmonic resonances, one can observe a clear splitting of the plasmonic mode, centered at the intersection of the MC absorption band and the plasmonic resonance in the SPY isomer. This anti-crossing behavior is a signature of a strong coupling regime. To support these experimental results, analytical calculations were made. The polarizability of the silver nanorod is calculated using a prolate spheroid in the dipole approximation:

[1]
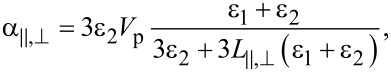


where 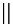 and 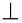 refer to the polarization of the incoming light, *V*_p_ is the volume of the particle, ε_1_ is silver permittivity, ε_2_ is the permittivity of the host medium, deduced by the ellipsometric measurements; while 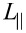 and 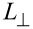 are geometrical factors given by:

[2]
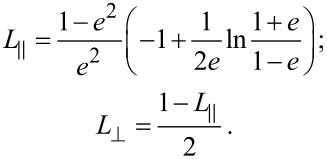


Here *e* is the eccentricity calculated as 
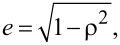
 with ρ the aspect ratio of the spheroid.

In order to account for the energy-shifting part of the retardation effect and the radiative loss, which are missing in the model, the equation was modified in a similar way as in [24] which led to Equation 3.

[3]
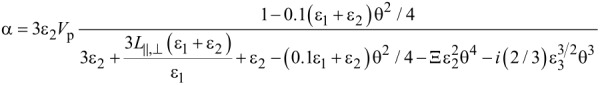


Here θ = 2πω/*c*, while Ξ = 1.4 is a phenomenological weighting factor we introduced to obtain an optimal fit of the experimental results.

The results are presented in Figure 3b and 3d for ellipsoids presenting a fixed 110 nm length and varying widths and for ellipsoids presenting a fixed 70 nm width and varying lengths, respectively. On each map, the dashed black curve represents the spectral position of the maximum of the extinction cross-section of the ellipsoids in the SPY medium (the corresponding maps are not shown here) and the horizontal dashed white line corresponds to the MC absorption band. On both maps, a clear anti-crossing is then observed when the black dashed curves cross the white dashed lines. Indeed, in Figure 3b, the anti-crossing appears for a rod width of 95 nm and in Figure 3d, it appears for a rod length of about 85 nm. These values are in agreement with the experimental values, specially knowing that the given geometrical parameters of the nanorods, mainly width and length, correspond to the designed values. Indeed, only few images have been recorded by electronic microscopy after the nanofabrication process and small variations between the designed and the real sizes can occur.

**Figure 4:**
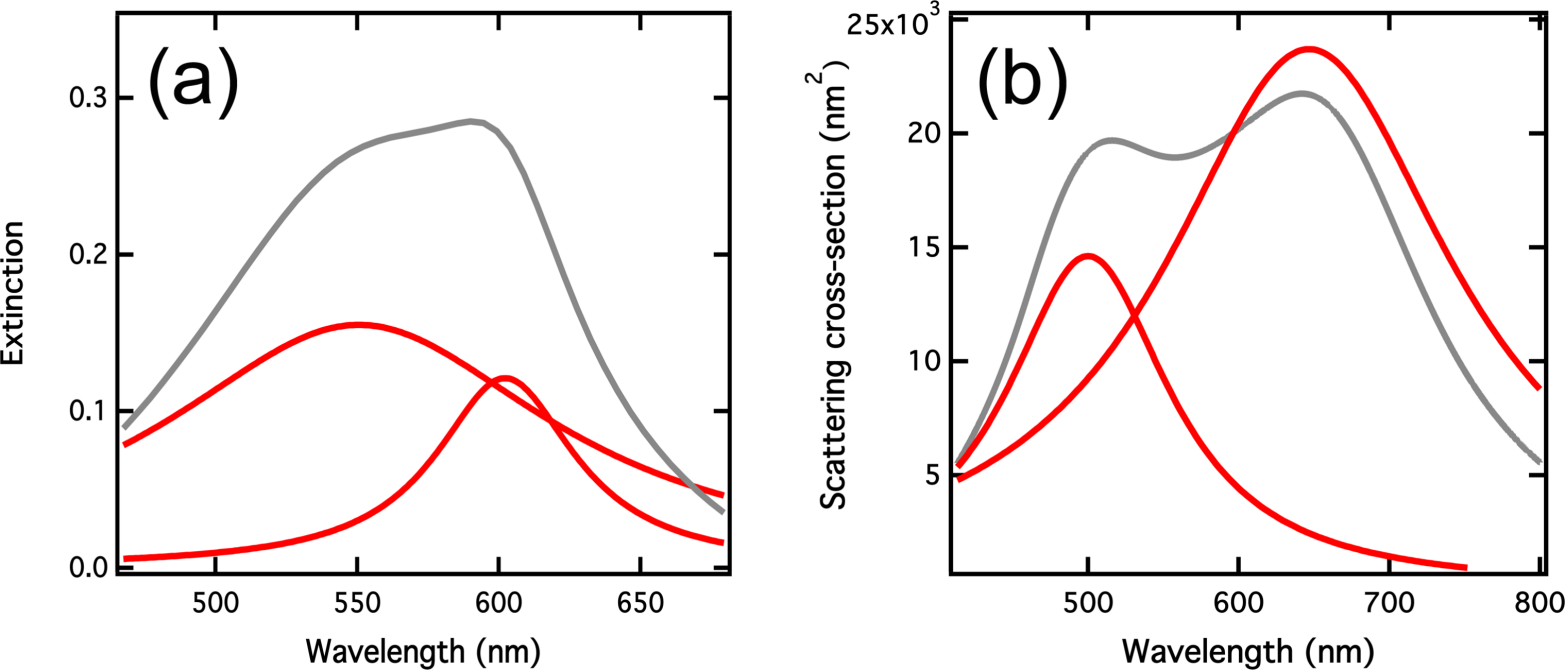
(a) Extinction spectrum (grey curve) recorded with a 70 nm wide and 90 nm long nanorod array covered with the MC isomer. The red curves correspond to the Lorentzian fitted peaks. (b) Extinction cross-section and the fitted peak decomposition, calculated for an ellipsoid of the same dimensions.

To confirm the observation of a strong coupling regime, we compared the linewidths of the hybrid modes obtained experimentally and theoretically for 70 nm wide and 90 nm long nanorods in the MC isomer (Figure 4). The incident polarization has been chosen to be aligned to the nanorod long axis (0°). Figure 4a shows the extinction spectrum (grey curve) recorded experimentally and the corresponding Lorentzian fitted peaks (red curves). The peaks are located at 551 and 602 nm and their spectral width are equal to 129 and 50 nm, leading to linewidths of 527 and 171 meV. Figure 4b shows the scattering cross-section spectrum obtained with our analytical model for a single ellipsoid in the MC isomer. The fitted peaks are located at 500 and 647 nm, indicating a Rabi splitting of 560 meV. This large value allowed us to conclude the observation of a strong coupling regime at the single nanorod level. Moreover, the spectral widths of the theoretical peaks are equal to 132 and 235 nm, leading to linewidths of 655 and 697 meV. This difference in the hybrid mode linewidths has already been observed for a gold nanogroove arrays coated with a J-aggregate dye film [25]. In this work, they observed different damping rates for the hybrid modes attributed to the interplay between the coherent dipole coupling between exciton and plasmon and the incoherent exchange of photon energy between both systems. This can happen when the damping of both separate oscillators is different, which is our case with the plasmonic resonance and the MC molecular exciton. The large linewidth difference we experimentally obtain for both hybrid modes is also probably due to the geometry dispersion in the nanorod array.

## Conclusion

In conclusion, we observed a polarization-dependent strong coupling between silver nanorods and the excited state of photochromic molecules. By properly designing the nanorods, the strong coupling can happen either with the longitudinal plasmonic mode, or with the transverse plasmonic mode, or even both. We also proved the existence of a strong coupling regime when the plasmonic resonance coincides with the molecular transition. Moreover, the calculations were in good agreement with our observations. Thus, we proved that the incident polarization allows for control of the plasmonic hybridization and the spectral position of the nanorod plasmon modes. This plasmonic hybridization is known to be reversible and can be enhanced by application of better resonators. As a perspective future application, this polarization-dependent strong coupling could be a prime interest for nanorods coupled to different emitters such as those in [17]. Indeed, in the anti-crossing region, the strong local field produced by the plasmonic resonance could be optically switched on and off, resulting in the controlled enhancement of the photoluminescence produced by the emitter.
